# Supplementary material for: Temporal Trend of Conventional Sperm Parameters in a Sicilian Population in the Decade 2011–2020
Source: J Clin Med. 2021 Mar 2;10(5):993. doi: 10.3390/jcm10050993 (PMC7957883; doi:10.3390/jcm10050993)
Supplement: Supplementary file 1 [file jcm-10-00993-s001.pdf]

Supplemetary Table S1. Summary of the studies evaluating the decline of sperm conventional parameters over time.

Author	Year	Study design	Country	WHO Manual	Number	Concentration	Total sperm count	Progressive motility	Total motility	Morphology	Other
Carlsen et al., [11]	1992	Review	-	NR	14,947	↓	↓	NA	NA	NA	NA
Auger et al., [1]	1995	Retrospectiv study	France	WHO 1992	1351	↓	NA	NA	↓	↓	NA
Adamopoulos et al., [2]	1996	Retrospective study	Greece	WHO 1992	2385	NA	↓	NA	NA	NA	NA
Van Waeleghem et al., [3]	1996	Retrospective study	Belgium	WHO 1987	416	↓	↔	↓	↓	↓	NA
Bujan et al., [16]	1996	Retrospective study	France	NR	302	↔	NA	NA	NA	NA	NA
Berling & Wölner-Hanssen [17]	1997	Retrospective study	Southern Sweden	WHO 1992	718	↑	NA	NA	↑	↑	↑ (base-value of the penetration test)
Rasmussen et al., [18]	1997	Retrospective study	Denmark	NR	1055	↔	NA	NA	NA	NA	NA
Emanuel et al., [19]	1998	Retrospective study	Minnesota	NR	374	↔	NA	NA	NA	NA	NA
Bilotta et al., [32]	1999	Review	Italy	NA	1068	↓	NA	NA	↓	↓	NA
Seo et al., [20]	2000	Retrospective study	Korea	WHO 1987	22249	↔	NA	NA	↔	NA	NA
Gandini et al., [24]	2000	Retrospective study	Italy	WHO 1982, 187, 1992	2471 (patients) 397 (donors)	↓ (patients) ↔ (donors)	↓ (patients) ↔ (donors)	↓ (patients) ↓ (donors)	↓ (patients) ↔ (donors)	NA	NA
Costello et al., [21]	2002	Retrospective study	Australia	WHO 1980, 1987, 1992	448	NA	↔	NA	↑	NA	NA
Marimuthu et al., [22]	2003	Retrospective study	India	NR	1176	NA	↔	NA	NA	NA	NA
Vicari et al., [33]	2003	Retrospective study	Italy	WHO 1999	716	↓	NA	NA	NA	↓	NA
Lackner et al., [5]	2005	Retrospective study	Austria	WHO 1987, 1992, 1999	7,780	↓	NA	NA	NA	NA	NA
Spirada et al., [6]	2007	Retrospective study	Northeast of Scotland	WHO 1993,1999	4832	↓	NA	↓	NA	NA	NA
Axelsson et al., [23]	2011	Cohort study	Sweden	WHO 1999	511	↔	↔	NA	↔	NA	NA
Elia et al., [34]	2012		Italy	WHO 1987, WHO 1999	1327	↑	↑	NA	NA	↑	NA
Geoffroy-Siraudin et al., [8]	2012	Retrospective study	France	WHO 1992, 2010	10 932	↓	↓	↓	NA	↓	NA

Rolland et al.,	2013	Retrospective study	France	NR	26,609	↓	NA	NA	↑	↓	
Mendiola et al., [10]	2013	Cross-sectional	Southern Spain	WHO 1999	215	NA	↓	NA	↔	↔	NA
Borges et al., [15]	2015	Retrospective study	Brazil	NR	2300	↓	↓	↔	NA	↓	NA
Levine et al., [26]	2017	Meta-regression study	North America, Europe, Australia, New Zealand	NR	42 935	↓	↓	NA	NA	NA	NA
Segupta et al. [27]	2017	Meta-regression study	Africa	NR	6767	↓	NA	NA	NA	NA	NA
Wang et al., [28]	2017	Retrospective study	China	WHO 1999	5210	↓	↓	↓	NA	NA	NA
Huang et al., [29]	2017	Retrospective study	China	WHO 1999, 2010	30,636	↓	↓	↓	NA	↓	NA
Basnet et al., [13]	2016	Retrospective study	North Norway	WHO,1987, 1992,2010	5739	↓	↓	NA	NA	NA	NA
Mishra et al., [14]	2018	Review	India	NR	13486	↓	NA	↔	↔	↓	NA
Sugihara et al., [12]	2020	Retrospective study	Belgium	WHO 1992, 1997, 2010	807	↔	↓	↔	↔	↓	↔ (clinical pregnancy rate)
Vahidi et al., [25]	2020	Retrospective study	Iran	WHO 1987, 1999	1815	↑	NA	NA	NA	↓	NA
Present study	2021	Retrospective study	Italy	WHO 2010	1409	↔	↓	↑	NA	↔	NA

Abbreviations. WHO, World Health Organization; NA, non assessed; NR, not reported.

↔, unchanged; ↓, reduced; ↑, increased.
